# Comment on “An Online Intervention Comparing a Very Low-Carbohydrate Ketogenic Diet and Lifestyle Recommendations Versus a Plate Method Diet in Overweight Individuals With Type 2 Diabetes: A Randomized Controlled Trial”

**DOI:** 10.2196/jmir.7672

**Published:** 2018-05-01

**Authors:** Andrew Nathan Reynolds

**Affiliations:** ^1^ Department of Human Nutrition University of Otago Dunedin New Zealand

**Keywords:** eHealth, diet, weight loss, type 2 diabetes mellitus, low-carbohydrate diets, type 2 diabetes

This letter is regarding the recent publication of “An Online Intervention Comparing a Very Low-Carbohydrate Ketogenic Diet and Lifestyle Recommendations Versus a Plate Method Diet in Overweight Individuals With Type 2 Diabetes: A Randomized Controlled Trial” [1]. The authors have been innovative in their use of an online platform, and present findings that are both interesting and relevant. The effect size of the intervention is considerable, highlighting the potential of lifestyle interventions delivered in this manner. However, the interpretation of the trial results requires further discussion.

The authors attribute the improvements in body weight and glycaemic control to a very low-carbohydrate ketogenic diet and lifestyle recommendations. To single out the very low-carbohydrate ketogenic diet recommendation above the other lifestyle recommendations made in the intervention is unsupported. The paper states that the self-reported dietary assessment method “should not be considered validated.” Furthermore, data to demonstrate dietary change due to the intervention, such as the measured urinary acetoacetate, has not been provided.

The observed effect of the intervention is at least as likely due to the other lifestyle recommendations, or recommendations acting in concert. Advice to increase physical activity [2] and sleep [3] as well as the provision of psychological support [4],  such as mindfulness training and goal setting, were included in the intervention and have been shown in meta analyses to independently improve the primary outcomes of the current paper. By contrast, a recent meta analysis of trials with type 2 participants randomised to lower or higher carbohydrate diets showed no difference in body mass index and blood glucose control (HbA_1c_) at 12 months, although there was a transient effect on HbA_1c_ at 3 and 6 months [5]. To further demonstrate the potentially unwarranted focus on the very low-carbohydrate ketogenic diet, the intervention of the current paper also included training on: awareness of hunger, fullness, craving, taste satisfaction and triggers for overeating, scheduling, noticing and savouring positive events, developing self-compassion, practicing positive reappraisal, gratitude, acts of kindness, and being aware of personal strengths. In contrast, the control group only received dietary advice. Given these disparities and the lack of supporting data it is unfeasible of the authors to attribute a change due to their recommendation to follow a very low-carbohydrate ketogenic diet.

Differences in outcomes between the intervention and control groups may further be attributed to the dissimilar level of participant support. Participants in the intervention group were contacted once a week for 16 weeks, and then once a fortnight for a further 16 weeks (24 times). The control group were contacted once a week for four weeks, and then once every four weeks (11 times). This disparity in the level of support and engagement is a probable driver for the participant drop out rate in the control (6 of 13) and intervention (1 of 12).

That the sample size is grossly inadequate is evident by differences in the baseline attributes of participants who, although randomised, were on average 18.8kg heavier and 5.2 years younger in the intervention group than those in the control group. An estimate for an adequate sample size would provide greater context to the results of this pilot study with the author’s previous work in this area providing them with the necessary data to do so. Finally, that change in body weight is the sole measure of body fatness is a further concern, with additional markers not confounded by baseline body weight required.

The findings of this study are undoubtedly interesting. The authors should be acknowledged for their work highlighting the potential of online interventions to deliver multiple lifestyle recommendations. The findings also suggest at the level of support required to enable behavioural change towards healthier behaviours. What the findings do not support is the promotion of very-low carbohydrate ketogenic diets.
